# Impact of a peer-counseling intervention on breastfeeding practices in different socioeconomic strata: results from the equity analysis of the PROMISE-EBF trial in Uganda

**DOI:** 10.3402/gha.v9.30578

**Published:** 2016-07-28

**Authors:** Kristiane Tislevoll Eide, Lars Thore Fadnes, Ingunn Marie Stadskleiv Engebretsen, Kristine Husøy Onarheim, Henry Wamani, James K. Tumwine, Ole Frithjof Norheim, for the PROMISE-EBF Study Group**

**Affiliations:** 1 Department of Global Public Health and Primary, Care, University of Bergen, Bergen, Norway; 2 Department of Clinical Dentistry, University of Bergen, Bergen, Norway; 3 Centre for International Health, Department of Global Public Health and Primary Care, University of Bergen, Bergen, Norway; 4 School of Public Health, Makerere University, Kampala, Uganda; 5 Department of Paediatrics and Child Health, Makerere University, Kampala, Uganda

**Keywords:** anthropometry, exclusive breastfeeding, infant health, socioeconomic factors, Uganda, undernutrition

## Abstract

**Background:**

Undernutrition is highly prevalent among infants in Uganda. Optimal infant feeding practices may improve nutritional status, health, and survival among children.

**Objective:**

Our study evaluates the socioeconomic distribution of exclusive breastfeeding (EBF) and growth outcomes among infants included in a trial, which promoted EBF by peer counselors in Uganda.

**Design:**

Twenty-four clusters comprising one to two communities in Uganda were randomized into intervention and control arms, including 765 mother-infant pairs (PROMISE-EBF trial, 200608, ClinicalTrials.gov no. NCT00397150). Intervention clusters received the promotion of EBF by peer counselors in addition to standard care. Breastfeeding and growth outcomes were compared according to wealth quintiles and intervention/control arms. Socioeconomic inequality in breastfeeding and growth outcomes were measured using the concentration index 12 and 24 weeks postpartum. We used the decomposition of the concentration index to identify factors contributing to growth inequality at 24 weeks.

**Results:**

EBF was significantly concentrated among the poorest in the intervention group at 24 weeks postpartum, concentration index −0.060. The control group showed a concentration of breastfeeding among the richest part of the population, although not statistically significant. Stunting, wasting, and underweight were similarly significantly concentrated among the poorest in the intervention group and the total population at 24 weeks, but showing non-significant concentrations for the control group.

**Conclusion:**

This study shows that EBF can be successfully promoted among the poor. In addition, socioeconomic inequality in growth outcomes starts early in infancy, but the breastfeeding intervention was not strong enough to counteract this influence.

## Introduction

Undernutrition is highly prevalent in low- and middle-income countries and is estimated to be responsible for 45% of all child deaths (1). A complex web of social, economic, and political factors influences child undernutrition, and there are substantial socioeconomic inequalities in undernutrition between and within populations. Early childhood undernutrition is further associated with poor child development, reduced school performance, and less productivity in adult life (1). The promotion of exclusive breastfeeding (EBF) for the first 6 months of infancy is suggested as an effective strategy to achieve better nutrition, lower infections, and increased intelligence (2–4). Hence, breastfeeding provides benefits beyond health and will provide economic and environmental advantages to women and children as well as the society at large (5). EBF can effectively be promoted by relevant interventions (5). However, more information is needed on how such strategies affect different socioeconomic strata.

A concern for policymakers is the health gap between different population groups (6, 7). There are unacceptably high levels of inequality in child health within and between countries, and in some areas, these gaps are widening (8–10). Victora et al. have called for a focus on equity in achieving the Millennium Development Goal targets and suggest that the first goal, poverty and hunger reduction, should be united with the fourth goal, reducing child mortality, to avoid making progress in child mortality rates at population levels while leaving the poorest children behind (10).

The concentration index has become a useful tool for measuring socioeconomic inequalities in health (11, 12). It can also be decomposed to investigate which factors are associated with inequality. The decomposition method has received considerable attention among health economists and epidemiologists (13–16), as it provides information on the determinants of inequalities.

### Setting

Uganda labors under a considerable burden of undernutrition, economic inequality, and poverty. In 2011, 28% of children were stunted, 4% of children were wasted, and 17% were underweight in the total population (17). Even for infants <6 months old, undernutrition was present, with a prevalence of stunting at 6.4%, wasting 5.0%, and underweight 4.4%. With a population of 37.6 million, the degree of income inequality in Uganda is relatively high (Gini coefficient of 44.3) (18). Approximately 38% of the population is living on <$1.25 per day (2005 international prices). Public health information may help to level some of these challenges. Our study addresses the possible benefits of such interventions on infant nutrition.

This study analyzes the Ugandan site of a community-based cluster-randomized trial promoting EBF for 6 months: the PROMISE-EBF study (ClinicalTrials.gov no. NCT00397150). Peer counselors provided breastfeeding support in the intervention clusters in addition to standard care. Mothers and infants in the control clusters received standard healthcare only. The prevalence of EBF at 12 weeks was doubled in the intervention clusters compared to the controls (19). No difference in the prevalence of diarrhea was noted at either 12 or 24 weeks. Growth outcomes at 24 weeks have been described elsewhere (20). There was a small, but insignificant, difference in length-for-age *z*-scores and slightly lower weight-for-length *z*-scores in the intervention group in Uganda. Population, health, and development indicators from Uganda and baseline characteristics from the study site are presented in Tables (1–3) (18–20).

**Table 1:** Population, health, and development indicators for Uganda

Characteristics	
Population indicators	
Total population, millions (2013)	37.6
Physicians per 10,000 people	1,2
Life expectancy at birth (2013)	59.2
Health indicators	
Total fertility rate	5.9
Maternal mortality ratio (per 100,000 live births)	310
Infant mortality rate (per 1,000 live births)	45
Under-five mortality rate (per 1,000 live births)	69
Immunization coverage, measles (% of 1 year olds)	82
Stunting under-five children (%)	33.4%
HIV prevalence females (% of 15–24-year olds)	4%
HIV prevalence males (% of 15–24-year olds)	2.3%
Development indicators	
GDP per capita (PPP $2005)	1,334
People living below PPP $1.25 a day (%)	38.01%
Health expenditure as % of GDP	9.5%
Human development index (HDI)	0.485
Income Gini coefficient	44.3
Adult literacy rate (15 years and above) (%)	73.2%

Source: From UNDP (18).

**Table 2:** Baseline characteristics of the study population in Uganda

	Intervention	Control
Categorical data		
Eligible mother–infant pairs	396	369
Marital status	392	366
Married	244 (62%)	234 (64%)
Cohabiting	119 (30%)	104 (28%)
Single, widowed, separated, or divorced	29 (7%)	28 (8%)
Socioeconomic-status quintile	396	369
1 (poorest)	91 (23%)	62 (17%)
2	97 (24%)	86 (23%)
3	76 (19%)	49 (13%)
4	71 (18%)	84 (23%)
5 (least poor)	61 (15%)	88 (24%)
Electricity in the house	391	361
Yes	53 (14%)	70 (19%)
Water source	392	363
Surface water and other	136 (35%)	97 (27%)
Borehole or tap	244 (62%)	246 (68%)
Piped yard or home	12 (3%)	20 (6%)
Toilet	339	328
None or open	84 (25%)	59 (18%)
Pit or ventilated improved pit	245 (72%)	266 (81%)
Flush	10 (3%)	3 (<1%)
Parity	392	366
Primipara	81 (21%)	85 (23%)
Multipara	311 (79%)	281 (77%)
Previous child death	307	277
Yes	109 (36%)	80 (29%)
Attendance at an antenatal clinic (index child)	376	352
Yes	272 (72%)	274 (78%)
Place of birth (index child)	381	351
Out of facility	208 (55%)	146 (42%)
Facility	173 (45%)	205 (58%)
Continuous data		
Maternal age	394	368
Years	25 (20–30)	24 (20–30)
Maternal education	391	365
Years	6 (4–8)	6 (5–9)
Maternal body mass index	343	312
6 weeks postpartum (kg/m^2^)	22 (20–24)	22 (20–24)
Monthly income	116	121
2007 euros	14 (5–28)	10 (5–23)

Data are number, number (%), or median (IQR).

Source: From Tylleskar et al. (19).

**Table 3:** Anthropometric characteristics of the study population in Uganda

Categorical data	Intervention	Control
Stunting (%)		
12 weeks postpartum	13.5	9.2
24 weeks postpartum	20.6	15.2
Wasting (%)		
12 weeks postpartum	6.1	3.2
24 weeks postpartum	7.6	3.2
Underweight (%)		
12 weeks postpartum	10.3	5.4
24 weeks postpartum	16.2	10.1

Source: From Engebretsen et al. (20).

The aim of this study was to investigate the EBF intervention's socioeconomic distribution and its impact on the socioeconomic distribution of undernutrition among infants included in the PROMISE-EBF trial in Uganda. Additionally, we investigate factors that contribute to inequality in early growth. We have not been able to identify any studies that evaluate inequalities in similar nutritional counseling interventions.

## Methods

### Data

The data were derived from the Ugandan site of the PROMISE-EBF study, which included 24 clusters in Mbale district, eastern Uganda, where 863 mothers were recruited and 765 mother−infant pairs enrolled for data collection. Pregnant women residing in the selected clusters intending to breastfeed were included in the study. All pregnant women in the clusters were identified by community-based recruiters and approached by the research team. Inclusion criteria were at least 7 months or visibly pregnant and not having given birth more than 1 week ago, providing informed consent, no plans of moving away the following year, and a singleton live birth without any malformation that could possibly obstacle breastfeeding. Exclusion criteria were women planning to replacement feed, giving birth more than 1 week before inclusion, and psychological illness (interfering with participation and consent). A visit was scheduled, and three attempts were made to visit mother–infant pairs. Data were collected between 2006 and 2008 with recruitment interviews late in pregnancy and scheduled visits at 3, 6, 12, and 24 weeks after birth. The recruitment interview gathered sociodemographic and socioeconomic information, while follow-up interviews gathered mother-reported feeding practices, infant illness, and anthropometric measurements. The clusters were randomized to intervention and control arms; randomization was stratified on urban (six clusters) and rural (18 clusters) characteristics. A cluster was a geographical area comprising one to two villages or communities with an average population of around 1,000 inhabitants. For further details on the intervention and main results, see Ref. (19).

### Variable definitions

#### Dependent variables

Infants classified as EBF did not receive any food or liquid other than breast milk (except for medication). This was reported at 12 and 24 weeks of age based on a 7-day recall (19).

Anthropometric assessment of nutritional status included length-for-age, weight-for-length, and weight-for-age, which can be expressed in standard deviation units, *z*-scores, from the median of a reference population. The WHO Child Growth Standards from 2006 were used as a reference population in this study to estimate the anthropometric *z*-scores of the infants examined (21). A child with a length-for-age *z*-score < –2 was considered stunted, a weight-for-length *z*-score < –2 was considered wasted, and a weight-for-age *z*-score < –2 was considered underweight. Means, means by quintiles and concentration indices for intervention and control arms, and the total population (Tables 4 and 5) were calculated on binary variables of *stunting*, *wasting*, and *underweight*. The secondary analysis of decomposing poor growth at 24 weeks to investigate factors contributing to inequality was based on the negative values of the *length-for-age z-score* at 24 weeks (explained under ‘Analysis’).

**Table 4:** Means by quintiles and concentration index of the EBF rate, stunting, wasting, and underweight at 12 weeks

12 weeks	Arm	*N*	Q1 (%)	Q2 (%)	Q3 (%)	Q4 (%)	Q5 (%)	Mean	Concentration index^a^	95% confidence interval	
EBF	Intervention	755	84.1	76.3	84.0	71.8	71.7	78.0	−0.018	−0.046	0.010
	Control	755	32.2	35.7	20.8	41.7	34.9	34.3	0.051	−0.027	0.130
	Total	755	61.4	56.8	58.4	55.5	49.0	57.0	−0.023	−0.056	0.011
Stunting	Intervention	642	17.1	14.1	16.2	12.0	9.3	14.1	−0.083	−0.223	0.058
	Control	642	13.7	10.0	12.4	8.3	4.3	9.3	−0.062	−0.256	0.133
	Total	642	15.7	12.2	14.7	10.0	2.3	11.8	−0.088	−0.201	0.026
Wasting	Intervention	639	9.9	5.9	4.6	5.1	7.6	6.7	−0.196	−0.439	0.048
	Control	639	5.6	1.4	2.7	2.7	3.0	3.0	−0.191	−0.561	0.179
	Total	639	8.2	3.8	3.9	3.8	5.0	4.9	**−0.213**	−0.416	−0.010
Underweight	Intervention	639	21.0	8.3	7.6	10.2	3.9	10.7	**−0.277**	−0.437	−0.116
	Control	639	13.4	2.8	7.5	1.3	2.9	4.9	−0.271	−0.551	0.009
	Total	639	17.9	5.7	7.6	5.3	3.4	8.0	**−0.301**	−0.438	−0.164

Statistically significant concentration indices reported in bold font.

a Concentration indices calculated on binary variables of stunting, wasting, and underweight.

Means for stunting, wasting, and underweight controlled for inverse probability weights. All means controlled for cluster correlation.

**Table 5:** Means by quintiles and concentration index of the EBF rate, stunting, wasting, and underweight at 24 weeks

24 weeks	Arm	N	Q1 (%)	Q2 (%)	Q3 (%)	Q4 (%)	Q5 (%)	Mean	Concentration index^a^	95% confidence interval	
EBF	Intervention	748	61.4	51.6	62.7	45.1	35.6	52.3	**−0.060**	−0.113	−0.006
	Control	748	10.2	13.1	8.2	13.4	10.5	11.4	0.049	−0.117	0.215
	Total	748	40.8	33.5	41.1	28.1	20.7	32.6	**−0.088**	−0.145	−0.032
Stunting	Intervention	639	32.5	16.8	22.9	17.6	13.2	21.2	**−0.135**	−0.249	−0.021
	Control	639	19.2	17.1	25.9	12.6	9.0	15.7	−0.077	−0.221	0.067
	Total	639	27.4	17.0	24.0	14.8	10.9	18.6	**−0.120**	−0.210	−0.031
Wasting	Intervention	639	18.3	7.5	3.0	7.4	5.7	8.9	−**0.236**	−0.423	−0.050
	Control	639	5.6	2.9	0^b^	0^b^	6.4	3.1	−0.152	−0.590	0.287
	Total	639	13.4	5.4	1.9	3.2	6.1	6.1	**−0.253**	−0.429	−0.078
Underweight	Intervention	641	28.9	17.2	15.2	12.1	9.4	17.4	**−0.221**	−0.342	−0.101
	Control	641	21.2	5.9	12.6	8.2	6.3	10.0	**−0.186**	−0.368	−0.003
	Total	641	25.9	12.0	14.2	10.0	7.7	14.0	**−0.226**	−0.327	−0.125

Statistically significant concentration indices reported in bold font.

a Concentration indices calculated on binary variables of stunting, wasting, and underweight.

b Omitted. Means for stunting, wasting and underweight controlled for inverse probability weights. All means controlled for cluster correlation.

#### Independent variables

Socioeconomic position was measured using data on assets and ownership obtained from household interviews and observations. A *wealth index* was created using multiple correspondence analysis (22), which is similar to analogous principal component analysis and suitable for categorical data. The index was based on the following assets and characteristics: TV, radio, mobile, chair, cupboard, refrigerator, type of toilet, electricity, type of water source, and type of wall. It was divided into five wealth *quintiles*. According to Wagstaff and Watanabe, it seems to make little difference in this kind of inequality analysis whether one measures socioeconomic position using an asset-based wealth index or household consumption (23).

Information on EBF included at most 765 mother–infant pairs, where missing, lost to follow-up, and deaths were recoded as non-events (not exclusively breastfeeding). Information on wealth status (index and quintiles) was available for 765 households collected in recruitment interviews. Anthropometric measurements were available for up to 691 mother–infant pairs, which were included in the analysis of inequality in growth. We included only ‘timely visits’ in our analysis. This included visits conducted in the following time periods: 9–18 weeks for the 12-week interview and 18–28 weeks for the 24-week interview. For information on the number of observations (*N*), see respective tables.

Explanatory variables included in the decomposition regression model were the wealth index, infant's *age* and *sex*, place of birth (at a *health facility (yes/no)*), *mothers’ education* in years, *marital status*, *residence* (rural/urban), participation in the breastfeeding *intervention*, *mother's age*, number of *previous births*, and *mother's height*. Due to missing values on any one of the explanatory variables, 126 observations were excluded in the regression analysis (*N*=565). The regression model allows us to explore the factors that are associated with poor growth and that contribute to the growth inequality.

### Analysis

All analyses were carried out in Stata IC 14 (www.stata.com). Concentration indices of EBF, stunting, wasting, and underweight at 12 and 24 weeks were computed using DASP Stata Package, a tool for distributive analysis (24). To adjust for missing anthropometric data, we used inverse probability weights when applicable (not in the inequality analysis) rather than plain available analysis to control for potential differences at follow-up. We also controlled for cluster correlation when possible. Further information on data cleaning and the handling of missing anthropometric information is available in earlier published data (20).

We used the concentration index as a measure of relative socioeconomic inequality in EBF, wasting, stunting, and underweight (11, 25). The concentration index ranges from −1 to 1, with the value 0 representing equality. The concentration index for a variable takes a negative value when the variable is more concentrated among the poorest of the population. Conversely, when a variable is concentrated among the least poor in the population, the concentration index is positive. For individual data, the concentration index can be written as follows (11):

1 $$\mathrm{C=}2/n\mathrm{\mu *}\sum (i=1{)}^{n}*y_{i}R_{i}-1$$

where *n* is the sample size, *y*_*i*_ is the health indicator for a person *i*, µ is the mean level of health in the sample, and *R*_*i*_ is the fractional rank of the *i*th person in terms of living standards.

Additionally, a concentration index for poor growth (using negative length-for-age *z*-scores) was further decomposed into possible factors that could explain inequality, a method proposed by Wagstaff et al. (15). We chose to decompose the concentration index for negative length-for-age *z*-scores (as a longitudinal outcome for poor growth) rather than a binary variable as stunting, as a continuous variable would keep more information in the linear regression model. This has also been the standard in other studies analyzing inequality in undernutrition using the decomposition method (11, 15, 16). For the outcome variable of poor growth: 1) a concentration index was calculated as a measure of socioeconomic inequality in health; 2) a multivariate regression model was estimated between the outcome variable and the set of explanatory variables; and 3) a decomposition of the inequality (concentration index) in the outcome variable was estimated to identify factors that contributed to inequality. For any linear regression model, the health outcome variable, *y*, can be linked to a set of *k* health determinants, *x*_*k*_, and an error term :

2 $$y_{i}=\alpha +\sum_{k}\beta_{k}x_{ki}+\varepsilon_{i}$$

Given this relationship between *y*_*i*_ and *x*_*ki*_, one is able to write the concentration index for *y* as follows (15):

3 $$C=\sum_{k}(\beta_{\text{k}}x_{k}/\mu )C_{k}+\text{G}{\text{C}}_{\varepsilon /\mu }=C_{y}+\text{G}{\text{C}}_{\varepsilon /\mu }$$

where the mean of *y* is written as µ, the mean of ${\bar{x}}_{k}$ is written as *x*_*k*_, and *C*_*k*_ is the concentration index for *x*_*k*_ (the socioeconomic inequality in each explanatory variable). The (${\text{β}}_{k}{\bar{x}}_{k}/\text{μ}$) is the elasticity of *y* (nutritional status) with respect to each ${\bar{x}}_{k}$ (explanatory variable). Elasticity means the (partial) association between a percentage change in the dependent health variable (nutritional status) and a percentage change in an explanatory variable, thereby indicating how responsive a change in nutritional status is to a change in, for example, mothers’ education. The elasticity is proportional with the beta coefficient from the multivariate regression model explaining the relationship between the explanatory variable *x*_*k*_ and the dependent variable *y*. The elasticity also adjusts for the mean of the dependent and explanatory variables. The last term is a residual (unexplained) component with a generalized concentration index, GC_ɛ_. This reflects the remaining inequality, which cannot be explained by the health determinants’ systematic variation across the socioeconomic rank. Each determinant's absolute contribution to *C* is the elasticity of the determinant multiplied by its concentration index ${\text{β}}_{k}{\bar{x}}_{k}/\text{μ}\text{*}C_{k}$ (13).

The decomposition method allows investigation into which factors contribute to inequality in health and how. There are two elements affecting whether a factor contributes to inequality in health: 1) the elasticity, which represents the association between the outcome variable and the explanatory variable, and 2) the degree of unequal distribution of the variable in the population (the explanatory variable's concentration index). A positive relative contribution implies that the variable has a supportive effect on the socioeconomic inequality in the health outcome. A negative relative contribution implies that the variable has the opposite effect on socioeconomic inequality in growth.

### Ethics statement

The PROMISE-EBF study was approved by the institutional review board of the Faculty of Medicine, Makerere University, and the Research and Ethics Committee, Uganda National Council for Science and Technology in Uganda, as well as the regional committees for Medical and Health Research Ethics in Norway. The women who participated in the peer-counseling program provided verbal-informed consent. Before data were gathered, a signed or thumb-printed informed consent was obtained.

## Results

The peer-counseling intervention increased EBF practices (52% of women in intervention clusters compared with 11% in control clusters at 24 weeks) (19). However, the intervention had a stronger impact on EBF at 24 weeks in the poorest three quintiles (58% compared to 11% in the control clusters). EBF was significantly concentrated among the poorest in the intervention group and the total population. For the control group, the concentration of EBF was among the wealthiest part of the population, but not statistically significant. There were no clear differences in EBF at 12 weeks between the socioeconomic strata (Table 4). The concentration index from week 12 showed a similar pattern to 24 weeks, but not statistically significant. Stunting, wasting and underweight were significantly conentrated among the poor in the total population and intervention group at 24 weeks. Similar distributions were present at 12 weeks for wasting and underweight. We found no significant difference in the socioeconomic distribution of stunting, wasting, or underweight between the intervention and control groups.

Results from the linear regression are presented in the left part of Table 6 (under the subheading adjusted linear regression). The beta coefficients show the association between the dependent variable (poor growth) and the explanatory variables. The dependent variable consists of negative values of length-for-age *z*-scores, representing poor growth. The higher the value, the poorer the growth. Therefore, a positive regression coefficient is interpreted as the variable being positively associated with undernutrition. Infants’ linear growth worsened with age (in weeks). Male infants were significantly more vulnerable to poor growth. Wealth was associated with better linear growth of infants, as was mothers’ height, indicating that shorter mothers were more likely to have children with poor growth.

**Table 6:** Decomposition of the concentration index for length-for-age *z*-scores <0

	Adjusted linear regression^a^				Inequality analysis			
		
Explanatory variables	β-coefficient	*P*	95% confidence interval		Elasticity	Concentration index	Absolute contribution	Relative contribution
Wealth index	**−0.089**	0.020	−0.163	−0.016	−0.104	0.39	−0.041	0.568
Infant age (weeks)	**0.078**	0.024	0.011	0.145	1.869	0.002	0.004	−0.049
Male infant	**1.108**	<0.001	0.930	1.285	0.537	−0.0004	−0.0002	0.003
Birth at facility	−0.081	0.290	−0.237	0.074	−0.041	0.142	−0.006	0.082
Mother's years of education	0.013	0.277	−0.011	0.036	0.077	0.108	0.008	−0.116
Mother is married or cohabiting	0.006	0.979	−0.429	0.440	0.005	−0.008	−0.00004	0.0005
Rural residence	0.006	0.953	−0.201	0.213	0.004	−0.099	−0.0004	0.006
Intervention arm^b^	0.155	0.101	−0.033	0.343	0.080	−0.066	−0.005	0.074
Number of births	0.015	0.449	−0.026	0.057	0.042	−0.033	−0.001	0.019
Mother's age	0.005	0.527	−0.011	0.020	0.121	0.006	0.0008	−0.011
Mother's height (in cm)	**−0.035**	<0.001	−0.049	−0.021	−5.317	0.002	−0.013	0.184
Residual (unexplained)							−0.017	0.24
Total							−0.071	1

*N*=565. Statistically significant associations reported in bold font for adjusted linear regression analysis.

a Controlled for cluster and inverse probability weights.

b Intervention promoting EBF through peer counseling.

The results from the decomposition are also presented in Table 6 (columns 5–8). The table presents the absolute and relative contributions of each explanatory factor to the total socioeconomic inequality in growth. The decomposition of the concentration index indicated 57% contribution from the wealth index to inequality. Mothers’ height contributed to 18% of the inequality, with a negative elasticity explaining the protective association to infant growth. Other factors contributed negatively to inequality (e.g. mothers’ education); hence, the contribution of all factors together accounts for 100% of the contribution. The infants’ age and sex, which show strong associations with linear growth, were not important in determining inequality in growth. A residual of 24% could not be explained by the factors included in this model.

## Discussion

This study has used concentration indices and decomposition to evaluate equity aspects in a trial promoting EBF with peer counselors. To the best of our knowledge, this method has rarely been used to evaluate a trial's effect on child health equity (26). Using this method in the assessment of the PROMISE-EBF trial showed that the trial outcome of EBF behavior change was achieved more successfully among the poorest than among the least poor. Thus, this intervention could counteract inequity. However, growth outcomes did not seem to go in the same direction. In contrast, the study indicates that socioeconomic inequality in infant nutrition adopted a pro-rich pattern as early as 12–24 weeks of age in spite of the changes in breastfeeding patterns. The inequality in linear growth was mainly associated with wealth status and mothers’ height.

EBF is highly recommended in low-income countries and has been suggested to prevent 800,000 annual child deaths with universal coverage (4). EBF provides benefits for children, such as fewer infections, increased intelligence, and perhaps less diabetes and overweight (4). There are many potential reasons that the pro-poor breastfeeding distribution did not achieve a less pro-rich distribution of undernutrition outcomes in this study. First, even if the recommended period of EBF is 6 months, the time for introducing other feeds is often best represented as a short interval ending around 6 months. In our study, the poorest women prolonged EBF more than the less poor, and several of them even beyond the recommended 6 months. The increased proportion of EBF among the poorest at 6 months confirms this trend. The mothers might not have had the resources to introduce complementary food even if the children gave signs indicating the need for additional food. For women who are financially better off, they might have planned for complementary feeding starting a bit earlier than 6 months and might already have increased caloric intake at the 6-month assessment. They might also have been enabled to feed themselves better and feed more frequently. The latter two issues were to a limited degree assessed in the trial (27). Moreover, EBF may not have the expected positive effect on infant growth at exactly 6 months’ age (20, 28, 29). Results derived from 15 randomized controlled trials show a small reduction in body mass index and bodyweight-for-length for infants at 6 months whose mothers had received a breastfeeding promotion intervention (4). More research is needed on this issue.

The decomposition analysis showed that male, older, and poorer infants were significantly more prone to poor growth. Our results also show that mothers’ height was highly associated with poor growth. This is in line with Barker et al. (30) and the extension of this hypothesis by Victora et al. (31), describing how maternal undernutrition influences fetal growth, which may lead to low birth weight, shortness, or thinness at birth as well as the failure of infant growth. Moreover, poor fetal growth or stunting during the first 2 years of life is associated with long-term consequences, such as shorter adult height, lower attainment in school, reduced income as an adult, and the reduced birthweight of future offspring. The association between mothers’ height and children's growth could be related by both hereditary and environmental factors. Further, the environmental factors could be linked to food security, which, most likely, is strongly correlated with wealth, family habits, and cultural practices, as well as other environmental aspects in the household.

In this study, we estimated a concentration index for stunting at −0.12 and wasting at −0.25 at 24 weeks. This shows somewhat more inequality than estimates from earlier multi-country reports; Gwatkin et al. estimated a concentration index for stunting in Uganda at −0.055 (32). Van de Poel et al.'s estimates on DHS data (UDHS 2000/01) presented concentration indices for stunting (−0.07) and wasting (0.01) (33). The figures in both studies are based on children younger than5 years, whereas our study only presents inequality among infants at 24 weeks. Our results suggest that inequality in undernutrition starts in early infancy. Compared with other regions of the world, sub-Saharan African and Southeast Asian countries have the highest prevalence of undernutrition, while it has been shown that the degree of inequality is lower than, for example, Latin American countries (33).

Analyses and results of the total study population require careful interpretation of causal inferences. As half of the participants were targets for the breastfeeding promotion intervention, how comparable is this group to the general population in Mbale? However, our analysis gave similar estimates of inequality in undernutrition for both the study arm in the breastfeeding intervention and the control group. Earlier analyses of the breastfeeding trial show changes in EBF practices, but not in the prevalence of diarrhea (19). Thus, we have results indicating self-reported behavioral change, but no or questionable change in health outcomes. Careful consideration of behavioral change interventions across socioeconomic strata is needed to have a more in-depth understanding in future attempts to promote safer infant feeding practices (34).

This study has several limitations. First, it is based on a population from Mbale district in eastern Uganda, which could only be generalizable to some extent to settings with similar wealth and culture. There is some diversity in wealth between areas in Uganda, and we know that Mbale is ranked somewhere in the middle. According to the UDHS from 2011, the Gini coefficient for economic inequality for the eastern region (0.35) is quite similar to that of the whole country (0.39) (17). Second, the number of observations is relatively small; hence, the study may not have had enough power to identify real differences between the intervention and control groups. We, therefore, cannot rule out a possible equity impact of the intervention. There was some loss to follow-up, and it is possible that those lost to follow-up were different from those retained. To account for this, we used inverse probability weight. When we analyzed the data both with and without applied weights (results not shown), we found little difference in the results, indicating the robustness of the analysis.

The intervention arm had an overall slightly lower socioeconomic status (SES) than the control arm. This was due to somewhat uneven distribution of socioeconomic factors at cluster randomization. One could therefore argue that the intervention arm showed a higher prevalence of EBF in the poorest quintiles due to a less-wealthy population in this arm. However, this small difference in SES between arms cannot explain the extensive difference we found in EBF practices between the intervention and control groups. And as we see in the linear regression in Table 6, being in the intervention arm had a non-significant impact on length-for-age *z*-scores, while the SES score was highly correlated with length-for-age *z*-scores. Hence, receiving peer counseling for EBF was less important for infant growth than the SES of the household.

Our findings indicate that wealth is a major contributor to inequality in undernutrition and that socioeconomic inequalities are present at very early ages. Therefore, poverty reduction is an important target in health planning and investment. Policymakers need to consider whether these health inequalities would best be addressed by focusing on the national healthcare system, poverty reduction, or both. Policies that promote growth on a macroeconomic level would probably not be sufficient alone, as they would only scratch the surface of an underlying social problem. Therefore, they should also encompass programs for poverty, unemployment, and inequality reduction (35). In some situations with vulnerable populations, such as in refugee camps, there seems to be a need for universal targeted nutritional interventions to achieve a well-nourished population, as argued by Briend et al. (36).

## Conclusion

This study evaluated equity aspects of a trial promoting EBF in Uganda and showed that EBF promotion by peer counselors can be effective when opting to influence feeding practices among the poorest. Although the outcome ‘EBF practice’ was more successfully achieved among the poorest than the wealthiest in the population, significant socioeconomic inequalities in stunting, wasting, and underweight were present as early as 24 weeks of infancy. There is a need to understand how to better deliver public health nutrition interventions to the poorest populations, and how to prevent unfavorable growth patterns. Community-based behavioral trials may be useful in reducing social inequalities in health, and this impact should be further explored. As better child growth and nutrition are related to numerous advantages in adult life, we suggest that improved equity will have potential advantages in future generations.

## Authors’ contributions

KTE, LTF, IMSE, KHO, HW, JKT, and OFN wrote the paper. Country PI-principal investigator in Uganda: JKT. KTE, LTF, IMSE, and OFN conceived of and designed this study. Data cleaning: HW, IMSE, LTF. KTE, IMSE and LTF analyzed the data. All authors read and approved the final manuscript.

## Notes

**The PROMISE-EBF Study Group

*Steering committee*: Thorkild Tylleskär^a^, Philippe Van de Perre^b,c,d^, Eva-Charlotte Ekström^e^, Nicolas Meda^f^, James K Tumwine^g^, Chipepo Kankasa^h^, Debra Jackson^i^

*Participating countries and investigators: Norway* Thorkild Tylleskär*^a^, Ingunn M S Engebretsen^a^, Lars T Fadnes^a^, Eli Fjeld Falnes^a^, Knut Fylkesnes^a^, Jørn I Klungsøyr^a^, Anne Nordrehaug-Åstrøm^j^, Bjarne Robberstad^a^, Halvor Sommerfelt^a^; *France* Philippe Van de Perre*^b,c,d^; *Sweden* Eva-Charlotte Ekström*^e^, Barni Nor^e^; *Burkina Faso* Nicolas Meda*^f^, Abdoulaye Hama Diallo^f^, Thomas Ouedrago^f^, Jeremi Rouamba^f^, Bernadette Traoré Germain Traoré^f^, Emmanuel Zabsonré^f^; *Uganda* James K Tumwine*^g^, Charles Karamagi^g^, Victoria Nankabirwa^j^, Jolly K Nankunda^g^, Grace Ndeezi^g^, Margaret Wandera^l^, Henry Wamani^i^; *Zambia* Chipepo Kankasa*^h^, Mary Katepa-Bwalya^h^, Chafye Siuluta^h^, Seter Siziya^h^; *South Africa* Debra Jackson^i^, Mickey Chopra^m^, Tanya Doherty^n^, Rebecca Shanmugam^n^, Ameena E Goga^n^, Carl Lombard^o^, Lungiswa Nkonki^n^, David Sanders^i^, Wesley Solomons^n^, Sonja Swanevelder^o^, Wanga Zembe^n^

*For every country, principal investigators are listed first; other authors are listed in alphabetical order of surname

- Centre for International Health, Department of Global Public Health and Primary Care University of Bergen, Norway
- INSERM U1058, Montpellier, France
- Université de Montpellier I, Montpellier, France
- Centre Hospitalier Universitaire Montpellier, Département de Bactériologie–Virologie, Montpellier, France
- Department of Women's and Children's Health, Uppsala University, Uppsala, Sweden
- MURAZ, Ministry of Health, Bobo-Dioulasso, Burkina Faso
- Department of Paediatrics and Child Health, College of Health Sciences, Makerere University, Kampala, Uganda
- Department of Paediatrics and Child Health, School of Medicine, University of Zambia, Lusaka, Zambia
- School of Public Health, University of Western Cape, Private Bag X17, South Africa
- Department of Clinical Dentistry, University of Bergen, Norway
- School of Public Health, College of Health Sciences, Makerere University, Kampala, Uganda
- Department of Dentistry, Makerere University, Makerere, Uganda
- UNICEF, UN Plaza, New York, NY, USA
- Medical Research Council, 7505 Cape Town, South Africa
- Biostatistics Unit, Medical Research Council, 7505 Cape Town, South Africa

## Paper context

Uganda labors under a considerable burden of undernutrition and poverty. Promotion of exclusive breastfeeding among the poor may improve this situation. Our study shows that promotion of exclusive breastfeeding by peer counselors is an effective strategy to increase breastfeeding among the poorest strata in a population. The study was not able to detect subsequent changes in infant growth. More research is needed regarding the effect of exclusive breastfeeding on infant growth.
